# Retraction Note: Factors affecting Nile crocodile *(Crocodylus niloticus)* leather quality: a systematic review

**DOI:** 10.1007/s11250-025-04708-w

**Published:** 2025-10-17

**Authors:** Ndzalo Khosa, Khetho Ratshilumela Nemutandani

**Affiliations:** Department of Agricultural Economics and Animal Production, University of Limpopo, Private Bag X1106, Sovenga, 0727 Limpopo South Africa

**Retraction Note: Tropical Animal Health and Production (2025) 57:126**

10.1007/s11250-025-04376-w

The Editor-in-Chief has retracted this article after concerns were raised regarding the validity of the content and certain references. Upon further investigation, the Publisher was unable to verify the source of references [Fuchs E (2006), Hutton J (1987), Manolis SC and Webb G (2005), Myburgh JG et al. (2020), Staton M and Doxson JR (1977), Swan GE et al. (2020)] and noted a mismatch between the title/named authors and the provided link for cited references [Ahmed M (2013), Fuchs E et al. (2019)]. References Veldsman DM et al. (2021) and Webb EC et al. (2021) are found to be identical.

The Editor-in-Chief therefore no longer has confidence in the reliability of this article.

All authors disagree with this retraction.

